# Comparison of Non-Contrast Coronary MRA Image Quality at 5T and 3T Based on the SCCT Segmental Model: A Technical Feasibility Study in Healthy Volunteers

**DOI:** 10.3390/jcm15093511

**Published:** 2026-05-04

**Authors:** Chuangwei Wei, Yan Xu, Runzhi Zhang, Wenjing Zhao, Nan Zhang, Jiayi Liu, Lei Xu, Zhaoying Wen

**Affiliations:** 1 Department of Radiology, Beijing Anzhen Hospital, Beijing Institute of Heart, Lung and Blood Vessel Disease, Capital Medical University, Beijing 100029, China; weichuangwei1205@163.com (C.W.); xuyan4458@163.com (Y.X.); zhangrz97@163.com (R.Z.); zhaowenjing2022@126.com (W.Z.); leixu2001@hotmail.com (L.X.); wenzhaoying11@163.com (Z.W.); 2 Department of Magnetic Resonance Imaging, Qinghai Provincial People’s Hospital, Xining 810000, China

**Keywords:** magnetic resonance angiography, coronary arteries, magnetic resonance imaging, ultra-high field, image quality

## Abstract

**Background**: This study aimed to evaluate the image quality of non-contrast-enhanced whole-heart coronary MR angiography (CMRA) using three different sequences: coronal-plane balanced turbo field echo (BTFE) at 3T, axial-plane modified Dixon (mDixon) at 3T, and axial-plane mDixon at 5T. **Methods**: Healthy young volunteers were prospectively enrolled from January 2025 to April 2025. Each participant underwent three CMRA scans—3T BTFE, 3T mDixon, and 5T mDixon—using customized MR protocols, all performed within 48 h. Subjective image quality was assessed based on the society of cardiovascular computed tomography 18-segment model using a four-point scale (1 = non-assessable to 4 = excellent). The assessability rate was defined as the percentage of segments receiving a score ≥ 2. Objective evaluation of the main coronary arteries included measurements of signal-to-noise ratio (SNR), contrast-to-noise ratio (CNR), vessel edge sharpness (VES), and visible vessel length. The Friedman test and one-way repeated measures analysis of variance (ANOVA) were performed to compare parameters obtained from 3T BTFE, 3T mDixon, and 5T mDixon. **Results**: A total of 20 participants (10 men; mean age, 24 ± 2 years) were included. Both 5T mDixon and 3T BTFE showed more favorable subjective image quality than 3T mDixon, particularly in distal and branch-level coronary segments. All three sequences achieved high vessel assessability. Quantitatively, 5T mDixon provided the highest SNR and CNR, while 3T BTFE showed the highest VES. Visible vessel lengths in LAD and RCA were longer with 5T mDixon and 3T BTFE versus 3T mDixon. However, 5T mDixon required the longest acquisition time (12.55 ± 2.80 min), consistent with its higher spatial resolution. **Conclusions**: In conclusion, in healthy volunteers, both 5T mDixon and 3T BTFE outperformed 3T mDixon in non-contrast CMRA, particularly in distal and branch-level coronary segments. While 5T mDixon provided the highest SNR and CNR, 3T BTFE achieved the greatest VES. These findings support the technical feasibility of both approaches, but further studies in patients are needed to confirm their clinical applicability

## 1. Introduction

Coronary artery disease (CAD) remains a leading cause of morbidity and mortality worldwide [1,2]. X-ray-based modalities remain the primary diagnostic approaches for identifying significant CAD, with invasive digital subtraction angiography (DSA) and non-invasive coronary computed tomography angiography (CCTA) being the most widely employed [3]. However, both techniques necessitate the use of iodinated contrast agents and entail patient exposure to ionizing radiation.

Non-contrast coronary Magnetic Resonance angiography (CMRA) represents a promising radiation-free and non-invasive alternative for assessing coronary artery stenosis, eliminating the need for iodinated contrast agents typically required in conventional imaging techniques [4,5,6,7]. Despite these advantages, the broader clinical adoption of non-contrast CMRA remains limited because image quality is highly susceptible to cardiac and respiratory motion, scan times are relatively long, distal coronary segments and branch vessels are often less well visualized, and performance may vary substantially depending on sequence design and field strength.

Among the sequences used for non-contrast CMRA, balanced steady-state free precession (bSSFP) has been widely adopted due to its inherently high blood signal and excellent blood-to-myocardium contrast [8,9]. In parallel, the modified Dixon (mDixon) water–fat separation technique has gained increasing application in coronary imaging, offering robust and homogeneous fat suppression owing to its reduced sensitivity to B_0_ inhomogeneities [10,11]. With ongoing advances in MR technology, ultra-high-field MR systems have increasingly been applied in cardiovascular imaging research. In theory, higher magnetic field strength substantially improves the signal-to-noise ratio (SNR), thereby providing a technical basis for achieving higher spatial resolution and superior image quality. However, ultra-high-field MR imaging also introduces several technical challenges, including exacerbated B_0_ and radiofrequency (B_1_) field inhomogeneities, increased sensitivity to motion artifacts, and elevated specific absorption rate (SAR). Against this background, the performance of imaging sequences across different field strengths has become a critical focus of investigation.

Recent studies have explored the optimization of these sequences under different imaging conditions. Lu et al. [12] demonstrated that the mDixon sequence at 5T significantly improved CMRA image quality, as evidenced by increased SNR, contrast-to-noise ratio (CNR), and vessel edge sharpness (VES) when compared to imaging at 3T. Additionally, a coronal-plane Balanced Turbo Field Echo (BTFE) sequence—the implementation of bSSFP—at 3T has been proposed, showing superior overall image quality relative to the conventional 3T mDixon sequence in non-contrast CMRA [13]. Although recent studies have reported encouraging results for individual CMRA implementations at 3T or 5T, direct intraindividual comparisons across field strengths and sequence types remain limited. In particular, a systematic comparison of 3T BTFE, 3T mDixon, and 5T mDixon based on the Society of Cardiovascular Computed Tomography (SCCT) 18-segment coronary artery model [14] has not been well established.

Therefore, the present study aimed to perform a systematic intraindividual comparison of three non-contrast CMRA approaches—axial 5T mDixon, axial 3T mDixon, and coronal 3T BTFE—in healthy volunteers using the SCCT 18-segment coronary artery model. By doing so, we sought to better characterize the respective effects of field strength and sequence type on CMRA image quality and to provide technical evidence for sequence selection and optimization across different MR platforms.

## 2. Materials and Methods

### 2.1. Participants

This study was approved by the Institutional Review Board of Beijing Anzhen Hospital, Capital Medical University, approval No. 2025232x. Written informed consent was obtained from all participants. From January 2025 to April 2025, 20 healthy young volunteers were prospectively enrolled.

Inclusion criteria were: (1) age between 18 and 35 years; (2) no known cardiovascular, respiratory, metabolic, or systemic disease; (3) no contraindications to MRI; and (4) healthy status confirmed by self-reported medical history and a brief clinical evaluation. Exclusion criteria included: (1) presence of cardiovascular risk factors (e.g., hypertension, diabetes); (2) history of congenital or acquired heart disease, prior cardiac surgery, or arrhythmia; and (3) inability to comply with MRI procedures.

A total of 20 healthy young volunteers were ultimately enrolled, with an equal sex distribution (10 men and 10 women). Their ages ranged from 21 to 28 years (mean age, 24 ± 2 years). All participants underwent non-contrast-enhanced, free-breathing, whole-heart CMRA. Healthy volunteers were intentionally selected because the present work was designed as a technical feasibility study aimed at comparing the image-quality performance of different CMRA approaches under relatively controlled conditions. This design helped minimize potential confounding effects of coronary calcification, luminal stenosis, arrhythmia, and other clinical factors that could independently affect image quality.

### 2.2. Image Acquisition

CMRA examinations were performed on a 3T MR scanner (Gyroscan Intera, Philips Medical Systems, Best, The Netherlands) with a 32-channel body coil and on a 5T MR scanner (uMR Jupiter, United Imaging Healthcare, Shanghai, China) using an 8-channel volume transmit coil in combination with 24-channel body and 48-channel spine receiver arrays. At 3T, T2 preparation was applied for both mDixon and BTFE acquisitions, while the 5T mDixon acquisition was performed without T2 preparation because this preparation module was not available in the current 5T implementation. Acquisition parameters for the 3T BTFE, 3T mDixon, and 5T mDixon sequences are summarized in Table 1. The scanning order of the three CMRA sequences (3T BTFE, 3T mDixon, and 5T mDixon) was randomly assigned for each participant to minimize sequence-order bias. All examinations were completed within a 48-h period, with the interval between scans determined by scanner availability. Participants were positioned supine, head-first, in the scanner. Survey images were acquired to locate the heart. The cine imaging in the four-chamber view was performed to determine the subject-specific trigger delay time and the optimal data acquisition window length, during which the motion of the right coronary artery was minimal. A beam-shaped respiratory navigator was positioned over the hemidiaphragm contralateral to the heart to compensate for respiratory motion. The 3T BTFE sequence was acquired in the coronal plane, while the 3T mDixon and 5T mDixon sequences were acquired in the axial plane. CMRA acquisitions were performed with free breathing. Because the CMRA protocols differed in spatial resolution and acquisition settings, scan duration varied accordingly. The 5T mDixon sequence used a smaller isotropic voxel size and higher spatial resolution, resulting in a longer acquisition time. Scan duration also depended on navigator efficiency. All 5T scans were performed under real-time safety monitoring of radiofrequency energy deposition, and no examination was interrupted because of SAR- or temperature-related concerns.

### 2.3. Imaging Analysis

All images were independently evaluated by two radiologists with extensive experience in cardiovascular imaging (N.Z. with 15 years and J.L. with 25 years). All CMRA datasets were anonymized before analysis and reviewed independently by two experienced radiologists in randomized order. The readers were blinded to participant identity, demographic information, and acquisition labels. Image post-processing and analysis were conducted on a Vitrea workstation (v8, Vital Images, Minnetonka, MN, USA). Image quality for all sequences was assessed using axial plane reconstructions.

In each participant, a total of 18 coronary artery segments were evaluated based on the Society of Cardiovascular Computed Tomography (SCCT) 18-segment model [14]. These included the left main coronary artery (LM); the proximal, mid, and distal segments of the left anterior descending artery (LAD-pro, LAD-mid, LAD-dis); the first and second diagonal branches (D1, D2); the proximal and distal segments of the left circumflex artery (LCX-pro, LCX-dis); the first and second obtuse marginal branches (OM1, OM2); the ramus intermedius (RI); the proximal, mid, and distal segments of the right coronary artery (RCA-pro, RCA-mid, RCA-dis); the left and right posterior descending arteries (L-PDA, R-PDA); and the left and right posterolateral branches (L-PLB, R-PLB). To ensure sufficient sample size for robust statistical analysis, certain small vessels present only in a subset of participants were grouped into broader categories: D1 and D2 were combined as D, OM1 and OM2 as OM, L-PDA and R-PDA as PDA, and L-PLB and R-PLB as PLB.

CMRA image quality of all 18 coronary artery segments was assessed using a four-point scale as follows [15,16]: 1 = non-assessable (severe artifacts, poor vessel contrast), 2 = assessable (moderate artifacts, fair vessel contrast), 3 = assessable (minor artifacts, good vessel contrast), 4 = assessable (no apparent artifacts, excellent vessel contrast). A segment was classified as non-assessable (score = 1) only when both readers reached consensus on its non-assessability. The assessability rate was defined as the proportion of coronary segments with image quality scores ≥ 2 (assessable) relative to the total number of segments analyzed.

SNR, CNR and Vessel edge sharpness (VES) were evaluated on axial-plane images at four predefined coronary locations: LM, LAD-pro, LCX-pro, and RCA-pro. At each site, a circular region of interest (ROI) measuring approximately 5 mm^2^ was manually placed at the center of the vessel lumen to determine the mean intraluminal blood signal intensity. A second ROI of similar size was positioned within the pericoronary adipose tissue at the same axial level to assess background noise. SNR was calculated as:

(1) $$SNR={SI}_{coronary\;artery}/{SD}_{pericoronary\;adipose},$$

where SI denotes the signal intensity within the coronary artery, and SD represents the standard deviation of signal intensity in the pericoronary adipose tissue. CNR was calculated as:

(2) $$CNR=({SI}_{coronary\;artery}-{SI}_{pericoronary\;adipose})/{SD}_{pericoronary\;adipose},$$

VES was quantified using a gradient-based algorithm implemented in MATLAB (R2019a; The MathWorks, Inc., Natick, MA, USA). Specifically, the VES was defined as the signal intensity change between 20% and 80% of the lumen-to-background profile divided by the corresponding spatial distance.

Curved planar reformation (CPR) was performed on the Vitrea post-processing workstation to process CMRA images. Subsequently, the visible segment lengths of LAD, LCX, and RCA were measured, among which the measurement of LAD length encompassed the LM segment.

The reproducibility of image analysis was evaluated through both intra- and interobserver assessments. For intraobserver analysis, one reader (N.Z.) reviewed all images twice, with a two-week interval between assessments to minimize bias. For interobserver analysis, a second reader (J.L.) independently evaluated the same CMRA datasets during the first measurement session conducted by the first reader, allowing for comparison of interobserver agreement.

### 2.4. Statistical Analysis

Data were tested for normality using the Shapiro-Wilk test. Variables with a normal distribution are presented as mean ± standard deviation, while non-normally distributed data are reported as median with interquartile range. Comparisons across the three imaging sequences (3T BTFE, 3T mDixon, and 5T mDixon) were performed using one-way repeated-measures analysis of variance (ANOVA) or the Friedman test, as appropriate. For normally distributed variables, one-way repeated measures ANOVA followed by post hoc Bonferroni correction was applied, and effect sizes were expressed as eta-squared (η^2^). For non-normally distributed variables, the Friedman test followed by pairwise Wilcoxon signed-rank tests with Bonferroni correction was used; effect sizes were reported as Kendall’s W and rank-biserial correlations, respectively. Effect sizes for post hoc Wilcoxon signed-rank tests were expressed as rank-biserial correlations and are provided in Supplementary Table S1. A post hoc sensitivity analysis using G*Power (version 3.1.9.7; Heinrich-Heine-Universität Düsseldorf, Düsseldorf, Germany) showed that, for the primary repeated-measures comparison across the three sequences, the present sample size (n = 20) was sufficient to detect an effect size of f = 0.295 or greater at α = 0.05 and power = 0.80. Interreader reproducibility for SNR, CNR, VES, and vessel length was assessed using intraclass correlation coefficients (ICC), with ICC values of 0.81–1.00 considered excellent. Interreader agreement for image quality scores was evaluated using Cohen’s kappa (κ), with κ ≥ 0.80 indicating excellent agreement. All analyses were performed using SPSS software (version 27.0; IBM Corp., Armonk, NY, USA), and all reported *p* values were Bonferroni-adjusted, with *p* < 0.05 considered statistically significant.

## 3. Results

### 3.1. Assessment of Image Quality

#### 3.1.1. Subjective Image Quality Score and Vessel Assessability Rate

**Figure 1:**
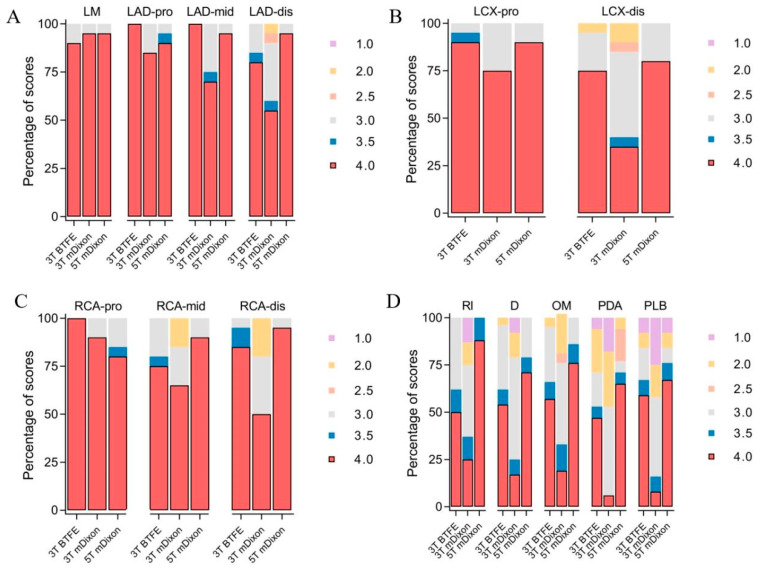
The stacked bar charts depict the distribution of subjective image quality scores (1–4) across 18 coronary segments for the three CMRA sequences (3T BTFE, 3T mDixon, and 5T mDixon). (**A**) Image quality scores for the LM, LAD-pro, LAD-mid, and LAD-dis; (**B**) Image quality scores for the LCX-pro and LCX-dis; (**C**) Image quality scores for the RCA-pro, RCA-mid, and RCA-dis; (**D**) Image quality scores for the RI, D, OM, PDA, and PLB. LM = left Main Artery, LAD-pro = proximal left anterior descending artery, LAD-mid = mid left anterior descending artery, LAD-dis = distal left anterior descending artery, LCX-pro = proximal left circumflex artery, LCX-dis = distal left circumflex artery, RCA-pro = proximal right coronary artery, RCA-mid = mid right coronary artery, RCA-dis = distal right coronary artery, D = the first and second diagonal branches (D1, D2), OM = the first and second obtuse marginal branches (OM1, OM2), RI = ramus intermedius, PDA = left and right posterior descending arteries (L-PDA, R-PDA), PLB = left and right posterolateral branches (L-PLB, R-PLB).

**Figure 2:**
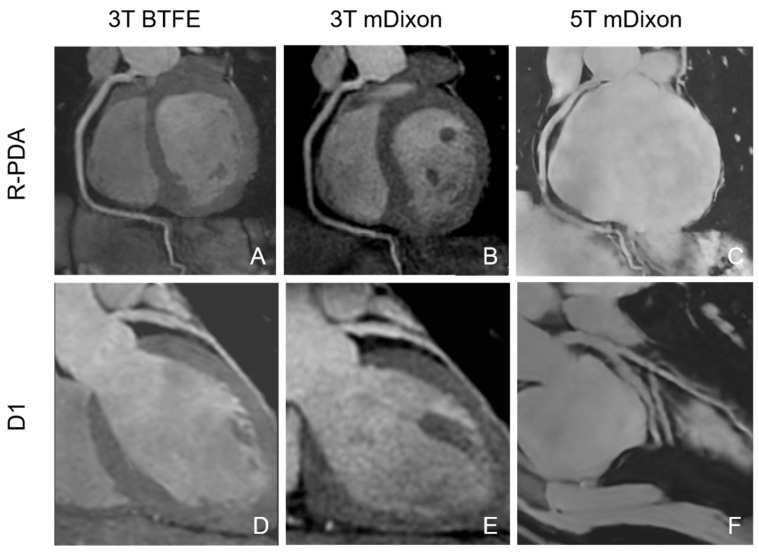
Noncontrast CMRA images show the PDA and D1 across the 3T BTFE, 3T mDixon and 5T mDixon sequences. (**A**) R-PDA on 3T BTFE; (**B**) R-PDA on 3T mDixon; (**C**) R-PDA on 5T mDixon; (**D**) D1 on 3T BTFE; (**E**) D1 on 3T mDixon; (**F**) D1 on 5T mDixon. R-PDA = right posterior descending artery, D1 = the first diagonal branch.

Subjective image quality scores for the three sequences are summarized in Table 2 and illustrated in Figure 1. Significant inter-sequence differences were observed in more distal or branch-level segments. Post hoc pairwise comparisons revealed that 5T mDixon and 3T BTFE consistently outperformed 3T mDixon in these more challenging segments. Subjective image quality scores were generally comparable between 5T mDixon and 3T BTFE, with significant differences observed only in the D and PDA segments. Representative noncontrast CMRA images of the D1 and PDA across the three sequences are shown in Figure 2. Effect size analysis using Kendall’s W showed the largest effect sizes observed in RI, D, OM, PDA, and PLB. Pairwise effect size estimates for post hoc comparisons are provided in Supplementary Table S1.

The assessability rates of coronary segments across the three sequences are summarized in Table 3. Differences among the three sequences were primarily observed in smaller distal and branch vessels. Overall, 5T mDixon demonstrated the highest assessability rate (99.6%), followed by 3T BTFE (99.2%) and 3T mDixon (96.2%). In branch-level coronary segments, both 5T mDixon and 3T BTFE consistently showed higher assessability than 3T mDixon, particularly in the PDA and PLB segments.

#### 3.1.2. Quantitative Image Quality Assessment

As shown in Table 4, 5T mDixon achieved the highest SNR and CNR across all coronary segments, followed by 3T BTFE, with 3T mDixon showing the lowest values (all *p* < 0.001). VES was highest with 3T BTFE, followed by 5T mDixon, and lowest with 3T mDixon (all *p* < 0.001). Effect size analysis showed large overall effects for these quantitative metrics, supporting substantial inter-sequence differences.

For visible vessel length, both 3T BTFE and 5T mDixon provided significantly longer LAD and RCA visualization than 3T mDixon. LCX length was not significantly different among the three sequences (3T BTFE: 78.3 ± 10.3 mm; 3T mDixon: 76.4 ± 9.1 mm; 5T mDixon: 78.0 ± 10.0 mm; *p* = 0.153), with a small effect size.

### 3.2. Agreements of Scoring and Measurements

Inter- and intraobserver reproducibility was excellent for all assessments across the 3T BTFE, 3T mDixon, and 5T mDixon sequences. For subjective image quality scores, Cohen’s κ values exceeded 0.80 for intraobserver agreement and were generally >0.80 for interobserver agreement. For quantitative image quality metrics (SNR, CNR, VES) and vessel length measurements, both intra- and interobserver ICCs were >0.90. Detailed κ and ICC values are summarized in Table 5 and Table 6.

## 4. Discussion

This study systematically compared 3T BTFE, 3T mDixon, and 5T mDixon sequences for non-contrast whole-heart CMRA. The main findings are summarized as follows: (a) In terms of subjective image quality, both 5T mDixon and 3T BTFE showed more favorable performance than 3T mDixon in distal and branch-level vessels. (b) 5T mDixon yielded the highest SNR and CNR, while 3T BTFE showed the greatest VES. (c) The visible lengths of the LAD and RCA were significantly longer with 5T mDixon and 3T BTFE than with 3T mDixon.

To the best of our knowledge, no prior study has performed a comprehensive segment-by-segment comparison of image quality across 3T BTFE, 3T mDixon, and 5T mDixon CMRA. In our cohort, both 5T mDixon and 3T BTFE achieved high subjective image quality and vessel assessability, achieving near-complete coverage across coronary segments, including the more challenging distal branches. The overall assessability rate was highest for 5T mDixon (99.6%), closely followed by 3T BTFE (99.2%), with both sequences maintaining 100% visualization in all major trunks and most small branches. Given that limited visualization of distal coronary segments remains a major obstacle to the broader clinical use of non-contrast CMRA, these findings suggest that both approaches may offer a technical means of improving distal coronary depiction. However, their value for stenosis assessment still requires validation in patient-based diagnostic studies.

Two recent studies support the findings of the present work. Yuan et al. [13] compared a 3T coronal-plane BTFE CMRA sequence provided superior image quality, including higher SNR, CNR, and VES, compared with 3T mDixon. Similarly, Lu et al. [12] reported significantly higher SNR, CNR, and VES at 5T than at 3T using the mDixon sequence. However, previous studies have generally focused on either sequence-based comparisons at a single field strength or field-strength–based comparisons using a single sequence. Direct comparisons of optimized CMRA strategies across different field strengths within the same volunteer cohort remain limited. In our study, 5T mDixon yielded the higher SNR and CNR, likely reflecting the inherent signal gain at higher field strength [17], whereas 3T BTFE maintained superior VES, attributable to the intrinsic advantages of SSFP sequences [9]. These findings highlight the complementary strengths of the two approaches—greater VES at 3T and enhanced SNR/CNR at 5T—and provide a technical basis for optimizing non-contrast CMRA across different field strengths. Nevertheless, the clinical significance of these quantitative improvements should be interpreted cautiously, as this study was not designed to determine whether they translate into improved diagnostic accuracy or clinical decision-making in patients with coronary artery disease.

In our present study, although 5T mDixon provided more favorable coronary-to-fat contrast compared with 3T mDixon, the coronary-to-myocardium contrast appeared less favorable, likely due to the routine use of a T2-preparation pulse in the 3T protocol. T2 preparation suppresses myocardial signal and thereby enhances coronary-to-myocardium contrast. Botnar et al. [18] demonstrated that incorporating T2 preparation into CMRA increased coronary-to-myocardium contrast by 123% and improved the delineation of the LAD and LCX by 33%, without measurable loss of SNR. This mechanism is particularly important in patients with myocardial bridging, where precise discrimination of intramyocardial coronary segments from adjacent myocardium is essential. At 5T, however, routine implementation of T2 preparation remains challenging because of technical constraints and the SAR burden with ultra-high-field imaging [19], which may partly explain the lower coronary-to-myocardium contrast observed in the present study.

The image quality advantages observed with the 5T mDixon sequence can be largely attributed to the inherent signal gain at ultra-high field strength. In our study, 5T CMRA was acquired using a significantly smaller isotropic voxel size (1.0 × 1.0 × 1.0 mm^3^) compared to 3T mDixon (1.5 × 1.5 × 1.5 mm^3^), which would typically result in a substantial reduction in SNR due to decreased voxel volume. However, despite this increase in spatial resolution, 5T mDixon achieved nearly double the SNR relative to 3T. For example, in the LM, the mean SNR increased from 46.85 at 3T to 88.65 at 5T, highlighting the substantial signal gain afforded by the ultra-high field. This signal advantage not only compensates for the expected SNR loss from smaller voxels but also enables improved delineation of vessel boundaries and more reliable visualization of distal coronary branches. While the higher spatial resolution and SNR came at the cost of prolonged scan time, the longer acquisition time should therefore be interpreted in the context of the substantial gains in spatial resolution and image quality, while also remaining a practical limitation for broader clinical implementation. These findings underscore the technical feasibility of 5T MRI for coronary imaging and support its further technical development, particularly when high spatial detail is required.

In our study, both 3T BTFE and 5T mDixon achieved significantly longer visible vessel lengths in the LAD and RCA compared to 3T mDixon, while no significant difference was observed in the LCX. This pattern may be explained by anatomical variation, as the LCX typically has a shorter course, whereas the LAD and RCA tend to extend further along the epicardial surface. The improved distal visualization achieved with 3T BTFE and 5T mDixon may therefore have contributed to the greater measured lengths of these vessels, in keeping with their more favorable image quality in distal segments.

It is also important to interpret the present findings within the context of current coronary imaging technologies. Photon-counting CT (PCCT) has recently demonstrated substantial advances in coronary imaging, including submillimeter spatial resolution, reduced radiation exposure compared with conventional CT, and high diagnostic accuracy relative to invasive angiography [20]. Compared with current PCCT, the present 5T CMRA approach still has important practical limitations, particularly its longer acquisition time (approximately 12 min) and lower spatial resolution (1.0 mm) than the submillimeter resolution currently achievable with PCCT. In this regard, the present study should not be interpreted as suggesting that non-contrast CMRA directly competes with or replaces contemporary CT-based coronary imaging in routine clinical practice. Rather, non-contrast CMRA and PCCT may be better viewed as complementary modalities with different strengths and clinical roles. While PCCT currently offers faster acquisition and superior spatial resolution, non-contrast CMRA may retain value in selected scenarios, such as younger individuals, subjects requiring radiation-free examinations, and patients with contraindications to iodinated contrast media.

The present findings should also be interpreted in light of the selected study population. Because all participants were healthy young volunteers, the observed feasibility and image quality may not be directly generalizable to patients with coronary artery disease, particularly those with arrhythmias or coronary calcification. CMRA acquisition in the present protocol was planned according to the relatively quiescent period of the right coronary artery, and image quality may therefore be more vulnerable to cardiac cycle irregularity in patients with arrhythmias. In addition, although prior work has suggested potential for 5T mDixon CMRA in coronary calcium detection [12], standardized criteria for coronary calcium quantification at 5T have not yet been established. Accordingly, the present results should be interpreted as technical feasibility data obtained under relatively controlled conditions, rather than as evidence of diagnostic applicability in calcified or diseased coronary arteries.

This study has several limitations. First, the sample size was relatively small and limited to a single center, which may affect the statistical power and generalizability of the findings. Second, only healthy young volunteers were included, thereby excluding elderly individuals or patients with known or suspected coronary artery disease. As a result, the generalizability of these findings to broader clinical populations remains limited. Third, although this study focused on image quality, no diagnostic accuracy analysis was performed using invasive angiography or CCTA as reference standards, limiting the ability to directly translate these results into clinical diagnostic performance. Fourth, despite anonymization and randomized image review, residual recognition bias cannot be completely excluded because the compared CMRA approaches differed in acquisition plane and image contrast characteristics. Future studies should include larger, demographically diverse cohorts across multiple centers and incorporate clinical reference standards, such as CCTA or invasive coronary angiography, to further assess diagnostic performance under real-world conditions. They should also evaluate advanced preparation strategies, including T2 preparation at 5T, and determine whether the observed image-quality advantages translate into measurable diagnostic benefit in patients with coronary artery disease.

## 5. Conclusions

In conclusion, both 5T mDixon and 3T BTFE demonstrated more favorable image-quality performance than 3T mDixon for non-contrast CMRA, particularly in distal and branch-level coronary segments. The 5T mDixon sequence provided the highest SNR and CNR, while 3T BTFE yielded the greatest VES. Both sequences yielded longer measurable vessel lengths, supporting their technical robustness under the present study conditions. These findings support the technical feasibility of 5T mDixon and 3T BTFE for non-contrast coronary imaging, while further validation in patient-based studies remains necessary.

## Figures and Tables

**Table 1:** Coronary Magnetic Resonance Angiography Acquisition Parameters.

Parameter	3T BTFE	3T mDixon	5T mDixon
No. slices	250	150	160
Field of view (mm^2^)	280 × 340	265 × 301	300 × 288
Acquired Matrix	208 × 234	176 × 201	300 × 288
TR (ms)	2.3	4.4	5.23
TE (ms)	1.13	1.42	1.85
Flip angle (degrees)	70	10	10
Fat/blood suppression	SPAIR	Water-fat-separation	Water-fat-separation
T2 preparation	40	30	no
Acquired voxel (mm^3^)	1.35 × 1.45 × 1.55	1.50 × 1.50 × 1.50	1.00 × 1.00 × 1.00
Slice thickness (mm)	1.55	1.50	1.00
Acquisition time (min)	6.08 ± 1.00	7.15 ± 1.70	12.55 ± 2.80

TR indicates repetition time; TE, echo time; SPAIR, spectrally selective adiabatic inversion recovery.

**Table 2:** Comparison of Subjective Image Quality Score at 3T BTFE, 3T mDixon, and 5T mDixon.

Characteristic	3T BTFE	3T mDixon	5T mDixon	Intergroups *p* Value *	*p* Value (Adjusted *p* Value) †	*p* Value (Adjusted *p* Value) ‡	*p* Value (Adjusted *p* Value) §	Effect Size (Kendall’s W)
Image quality score								
LM	4.0 (4.0–4.0)	4.0 (4.0–4.0)	4.0 (4.0–4.0)	0.779	n/a	n/a	n/a	0.000
LAD-pro	4.0 (4.0–4.0)	4.0 (4.0–4.0)	4.0 (4.0–4.0)	0.247	n/a	n/a	n/a	0.088
LCX-pro	4.0 (4.0–4.0)	4.0 (3.3–4.0)	4.0 (4.0–4.0)	0.276	n/a	n/a	n/a	0.064
RCA-pro	4.0 (4.0–4.0)	4.0 (4.0–4.0)	4.0 (4.0–4.0)	0.05	n/a	n/a	n/a	0.100
LAD-mid	4.0 (4.0–4.0)	4.0 (3.1–4.0)	4.0 (4.0–4.0)	0.006	0.060	0.951	0.102	0.258
RCA-mid	4.0 (3.6–4.0)	4.0 (3.0–4.0)	4.0 (4.0–4.0)	0.036	0.357	0.306	0.033	0.350
LAD-dis	4.0 (4.0–4.0)	4.0 (3.0–4.0)	4.0 (4.0–4.0)	0.005	0.048	1.000	0.030	0.218
LCX-dis	4.0 (3.3–4.0)	3.0 (3.0–4.0)	4.0 (4.0–4.0)	0.004	0.042	1.000	0.033	0.281
RCA-dis	4.0 (4.0–4.0)	4.0 (3.0–4.0)	4.0 (4.0–4.0)	0.001	0.033	1.000	0.030	0.338
RI	3.75(3.0–4.0)	3.0 (2.3–3.9)	4.0 (4.0–4.0)	0.005	0.123	0.189	0.081	0.661
D	4.0 (3.0–4.0)	3.0 (3.0–3.8)	4.0 (3.6–4.0)	<0.001	0.009	0.03	<0.001	0.540
OM	4.0 (3.0–4.0)	3.0 (2.0–3.8)	4.0 (3.5–4.0)	<0.001	˂0.001	0.294	˂0.001	0.551
PDA	3.5(2.0–4.0)	3.0 (2.0–3.0)	4.0 (2.8–4.0)	<0.001	0.021	0.048	˂0.001	0.642
PLB	4.0 (3.0–4.0)	3.0 (1.3–3.0)	4.0 (3.1–4.0)	<0.001	0.021	1.000	0.033	0.624

* The Friedman test was used to compare characteristics among 3T BTFE, 3T mDixon, and 5T mDixon. † 3T BTFE versus 3T mDixon. ‡ 3T BTFE versus 5T mDixon. § 3T mDixon versus 5T mDixon. Note: D = D1 + D2; OM = OM1 + OM2; PDA = L-PDA + R-PDA; PLB = L-PLB + R-PLB. “n/a” indicates not applicable.

**Table 3:** Comparison of Vessel Assessability Rates of Whole-Heart CMRA Sequences at 3T BTFE, 3T mDixon and 5T mDixon in 20 Participants.

Segment	No. of All Segments	No. of Assessable Segment (Score ≥ 2)		
		BTFE *n* (%)	3T mDixon *n* (%)	5T mDixon *n* (%)
Overall	262	260 (99.2)	252 (96.2)	261 (99.6)
LM	20	20 (100)	20 (100)	20 (100)
LAD-pro	20	20 (100)	20 (100)	20 (100)
LCX-pro	20	20 (100)	20 (100)	20 (100)
RCA-pro	20	20 (100)	20 (100)	20 (100)
LAD-mid	20	20 (100)	20 (100)	20 (100)
RCA-mid	20	20 (100)	20 (100)	20 (100)
LAD-dis	20	20 (100)	20 (100)	20 (100)
LCX-dis	20	20 (100)	20 (100)	20 (100)
RCA-dis	20	20 (100)	20 (100)	20 (100)
RI	8	8 (100)	7(87.5)	8(100)
D	24	24 (100)	22 (91.7)	24 (100)
OM	21	21 (100)	20 (95.2)	21 (100)
PDA	17	16 (94.1)	14 (82.4)	17 (100)
PLB	12	11 (91.7)	9 (75)	11 (91.7)

Note: D = D1 + D2; OM = OM1 + OM2; PDA = L-PDA + R-PDA; PLB = L-PLB + R-PLB.

**Table 4:** Quantitative Image Quality Metrics for 3T BTFE, 3T mDixon and 5T mDixon Sequences.

Characteristic	3T BTFE	3T mDixon	5T mDixon	Intergroups *p* Value *	*p* Value (Adjusted *p* Value) †	*p* Value (Adjusted *p* Value) ‡	*p* Value (Adjusted *p* Value) §	Effect Size (Eta-Squared)
SNR								
LM	65.35 ± 9.31	46.85 ± 10.72	88.65 ± 10.0	˂0.001	˂0.001	˂0.001	˂0.001	0.818
LAD_pro	66.70 ± 9.15	45.05 ± 13.24	89.85 ± 10.51	˂0.001	˂0.001	˂0.001	˂0.001	0.826
LCX_pro	68.70 ± 12.43	41.70 ± 11.18	89.90 ± 14.08	˂0.001	˂0.001	˂0.001	˂0.001	0.823
RCA_pro	63.30 ± 13.84	38.75 ± 9.40	84.30 ± 9.25	˂0.001	˂0.001	˂0.001	˂0.001	0.824
CNR								
LM	64.75 ± 9.56	40.20 ± 9.77	86.95 ± 10.87	˂0.001	˂0.001	˂0.001	˂0.001	0.842
LAD_pro	64.35 ± 9.46	39.00 ± 12.03	87.25 ± 11.15	˂0.001	˂0.001	˂0.001	˂0.001	0.848
LCX_pro	65.95 ± 12.39	39.95 ± 10.58	87.10 ± 13.94	˂0.001	˂0.001	˂0.001	˂0.001	0.842
RCA_pro	59.30 ± 12.16	33.30 ± 9.09	82.20 ± 8.76	˂0.001	˂0.001	˂0.001	˂0.001	0.857
VES								
LM	263.0 ± 30.14	81.80 ± 14.98	190.8 ± 16.93	˂0.001	˂0.001	˂0.001	˂0.001	0.966
LAD_pro	273.3 ± 34.72	82.75 ± 13.21	188.1 ± 16.69	˂0.001	˂0.001	˂0.001	˂0.001	0.990
LCX_pro	281.3 ± 31.67	74.30 ± 7.90	172.1 ± 18.27	˂0.001	˂0.001	˂0.001	˂0.001	0.967
RCA_pro	290.0 ± 35.69	78.45 ± 9.76	183.4 ± 12.47	˂0.001	˂0.001	˂0.001	˂0.001	0.961
length (mm)								
LAD	128.7 ± 2.2	115.0 ± 3.8	127.4 ± 2.0	˂0.001	0.003	1.00	0.027	0.316
LCX	78.3 ± 10.3	76.4 ± 9.1	78.0 ± 10.0	0.153	0.179	1.00	1.00	0.043
RCA	122.9 ± 1.6	120.9 ± 1.7	122.6 ± 1.7	˂0.001	˂0.001	1.00	0.02	0.398

* The Friedman test was used to compare characteristics among 3T BTFE, 3T mDixon, and 5T mDixon. † 3T BTFE versus 3T mDixon. ‡ 3T BTFE versus 5T mDixon. § 3T mDixon versus 5T mDixon. Note: D = D1 + D2; OM = OM1 + OM2; PDA = L-PDA + R-PDA; PLB = L-PLB + R-PLB.

**Table 5:** Inter- and Intraobserver Agreements in the Scoring of Image Quality among Three Different Sequences.

Score of Vessel Segments	Reader 1	Reader 2	Interobserver Kappa (95%CI)	Reader 1 Repeat	Intraobserver Kappa (95%CI)
LM	4.0 (4.0–4.0)	4.0 (4.0–4.0)	0.880 (0.648, 1.112)	4.0 (4.0–4.0)	1.00 (1.00, 1.00)
LAD_pro	4.0 (4.0–4.0)	4.0 (4.0–4.0)	0.883(0.648, 1.112)	4.0 (4.0–4.0)	0.880 (0.648, 1.112)
LCX_pro	4.0 (4.0–4.0)	4.0 (4.0–4.0)	0.870 (0.693, 1.046)	4.0 (4.0–4.0)	0.932 (0.799, 1.064)
RCA_pro	4.0 (4.0–4.0)	4.0 (4.0–4.0)	0.815 (0.568, 1.063)	4.0 (4.0–4.0)	0.900 (0.707, 1.093)
LAD_mid	4.0 (4.0–4.0)	4.0 (4.0–4.0)	0.815 (0.568, 1.063)	4.0 (4.0–4.0)	0.914 (0.747, 1.081)
RCA_mid	4.0 (4.0–4.0)	4.0 (4.0–4.0)	0.920 (0.807, 1.032)	4.0 (4.0–4.0)	0.962 (0.889, 1.036)
LAD_dis	4.0 (4.0–4.0)	4.0 (4.0–4.0)	0.835 (0.673, 0.998)	4.0 (4.0–4.0)	0.871 (0.736, 1.007)
LCX_dis	4.0 (3.0–4.0)	4.0 (3.0–4.0)	0.883 (0.773, 0.993)	4.0 (3.0–4.0)	0.940 (0.861, 1.020)
RCA_dis	4.0 (4.0–4.0)	4.0 (4.0–4.0)	0.819 (0.662, 0.976)	4.0 (4.0–4.0)	0.925 (0.820, 1.030)
RI	4.0 (3.0–4.0)	4.0 (3.0–4.0)	0.764 (0.516, 1.011)	4.0 (3.0–4.0)	0.922 (0.626, 1.030)
D	4.0 (3.0–4.0)	3.0 (3.0–4.0)	0.841 (0.734, 0.947)	3.5 (3.0–4.0)	0.882 (0.786, 0.978)
OM	3.5(3.0–4.0)	3.0 (3.0–4.0)	0.802 (0.650, 0.955)	3.0 (3.0–4.0)	0.887 (0.777, 0.996)
PDA	3.0 (2.0–4.0)	3.0 (2.0–4.0)	0.903 (0.818, 0.987)	3.0 (2.0–4.0)	0.906 (0.824, 0.987)
PLB	4.0 (2.3–4.0)	3.0 (2.0–4.0)	0.902 (0.805, 0.998)	3.0 (2.3–4.0)	0.925 (0.840, 1.011)

Note: D = D1 + D2; OM = OM1 + OM2; PDA = L-PDA + R-PDA; PLB = L-PLB + R-PLB.

**Table 6:** Inter- and Intraobserver Agreements in the Quantitative Image Quality and Vessel Length among Three Different Sequences.

	Reader 1	Reader 2	Interobserver ICC (95%CI)	Reader 1 Repeat	Intraobserver ICC (95%CI)
SNR					
LM	66.65 ± 19.97	67.77 ± 21.41	0.985 (0.974, 0.991)	67.25 ± 19.81	0.995 (0.991, 0.997)
LAD_pro	66.37 ± 21.17	68.18 ± 22.05	0.979 (0.966, 0.988)	67.53 ± 21.90	0.993 (0.988, 0.996)
LCX_pro	66.18 ± 23.14	66.55 ± 22.08	0.962 (0.937, 0.977)	66.12 ± 23.06	0.977 (0.966, 0.982)
RCA_pro	62.15 ± 21.80	63.32 ± 21.79	0.956 (0.927, 0.973)	61.58 ± 21.94	0.968 (0.947, 0.981)
CNR					
LM	63.09 ± 20.68	65.95 ± 24.26	0.965 (0.942, 0.979)	64.25 ± 22.51	0.982 (0.971, 0.989)
LAD_pro	62.77 ± 21.82	66.27 ± 26.08	0.948 (0.916, 0.968)	63.92 ± 23.52	0.985 (0.976, 0.991)
LCX_pro	62.57 ± 23.81	65.17 ± 24.62	0.957 (0.930, 0.974)	65.65 ± 22.06	0.990 (0.982, 0.994)
RCA_pro	57.35 ± 21.50	60.05 ± 25.43	0.968 (0.947, 0.981)	58.55 ± 23.31	0.984(0.973, 0.990)
VES					
LM	174.87 ± 74.80	182.38 ± 84.00	0.988(0.981, 0.993)	181.53 ± 81.63	0.991(0.985, 0.995)
LAD_pro	177.68 ± 78.59	185.20 ± 87.75	0.989(0.982, 0.994)	184.35 ± 85.43	0.992(0.987, 0.995)
LCX_pro	177.62 ± 84.92	181.27 ± 89.57	0.969(0.949, 0.982)	174.12 ± 91.02	0.992(0.987, 0.995)
RCA_pro	181.70 ± 86.99	183.75 ± 90.62	0.991(0.985, 0.995)	186.20 ± 92.79	0.997(0.995, 0.998)
length (mm)					
LAD	122.58 ± 13.66	124.02 ± 15.05	0.980 (0.967, 0.988)	124.60 ± 13.73	0.996(0.994, 0.998)
LCX	76.97 ± 9.50	78.13 ± 10.27	0.954(0.924, 0.972)	77.47 ± 10.13	0.968(0.947, 0.981)
RCA	121.95 ± 7.33	123.48 ± 7.54	0.971(0.953, 0.983)	121.90 ± 7.51	0.985(0.976, 0.991)

## Data Availability

The raw imaging data are not publicly available at this stage because they are being used for additional ongoing analyses and planned secondary manuscripts. The de-identified secondary measurement dataset and analysis code supporting the findings of this study are available from the corresponding author upon reasonable request.

## Supplementary Materials

The following supporting information can be downloaded at: https://www.mdpi.com/article/10.3390/jcm15093511/s1, Table S1: Rank-biserial correlations for post hoc pairwise Wilcoxon signed-rank comparisons of subjective image quality scores across the three CMRA sequences.
