# Supplementary material for: Comparison of Non-Contrast Coronary MRA Image Quality at 5T and 3T Based on the SCCT Segmental Model: A Technical Feasibility Study in Healthy Volunteers
Source: J Clin Med. 2026 May 4;15(9):3511. doi: 10.3390/jcm15093511 (PMC13164151; doi:10.3390/jcm15093511)
Supplement: Supplementary file 1 [file jcm-15-03511-s001.zip › jcm-4218306-supplementary.pdf]

Supplementary Table S1. Rank-biserial correlations for post hoc pairwise Wilcoxon signed-rank comparisons of subjective image quality scores across the three CMRA sequences.

Segment	3T BTFE vs 3T mDixon	3T mDixon vs 5T BTFE	5T mDixon vs 3T mDixon
LM	0.3300	0.3300	0.0000
LAD-pro	-1.0000	0.3300	-1.0000
LAD-mid	-1.0000	1.0000	-1.0000
LAD-dis	-0.8700	0.6900	0.3000
LCX-pro	-0.8700	0.6000	-0.2000
LCX-dis	-0.7900	0.8300	0.2500
RCA-pro	-1.0000	0.0000	-1.0000
RCA-mid	-1.0000	1.0000	0.0000
RCA-dis	-1.0000	1.0000	0.3000
RI	-1.0000	1.0000	1.0000
D	-0.7700	0.9800	1
OM	-0.9800	1.0000	0.8000
PDA	-0.8500	1.0000	1.0000
PLB	-1.0000	1.0000	0.3300

LM = left Main Artery, LAD-pro = proximal left anterior descending artery, LAD-mid = mid left anterior descending artery, LAD-dis = distal left anterior descending artery, LCX-pro = proximal left circumflex artery, LCX-dis = distal left circumflex artery, RCA-pro = proximal right coronary artery, RCA-mid = mid right coronary artery, RCA-dis = distal right coronary artery, D = the first and second diagonal branches (D1, D2), OM = the first and second obtuse marginal branches (OM1, OM2), RI = ramus intermedius, PDA = left and right posterior descending arteries (L-PDA, R-PDA), PLB = left and right posterolateral branches (L-PLB, R-PLB).
